# Leukotriene B4 receptor 2 governs macrophage migration during tissue inflammation

**DOI:** 10.1016/j.jbc.2023.105561

**Published:** 2023-12-12

**Authors:** Ebru Ermis, Titli Nargis, Kierstin Webster, Sarah A. Tersey, Ryan M. Anderson, Raghavendra G. Mirmira

**Affiliations:** 1 Kovler Diabetes Center, The University of Chicago, Chicago, Illinois, USA; 2 The College, The University of Chicago, Chicago, Illinois, USA; 3 Department of Medicine, The University of Chicago, Chicago, Illinois, USA; 4 Department of Pediatrics, The University of Chicago, Chicago, Illinois, USA

**Keywords:** macrophage, G-protein coupled receptor (GPCR), inflammation, zebrafish, mouse, metabolism

## Abstract

Chronic inflammation is the underlying cause of many diseases, including type 1 diabetes, obesity, and non-alcoholic fatty liver disease. Macrophages are continuously recruited to tissues during chronic inflammation where they exacerbate or resolve the pro-inflammatory environment. Although leukotriene B4 receptor 2 (BLT2) has been characterized as a low affinity receptor to several key eicosanoids and chemoattractants, its precise roles in the setting of inflammation and macrophage function remain incompletely understood. Here we used zebrafish and mouse models to probe the role of BLT2 in macrophage function during inflammation. We detected BLT2 expression in bone marrow derived and peritoneal macrophages of mouse models. Transcriptomic analysis of *Ltb4r2−/−* and WT macrophages suggested a role for BLT2 in macrophage migration, and studies *in vitro* confirmed that whereas BLT2 does not mediate macrophage polarization, it is required for chemotactic function, possibly mediated by downstream genes *Ccl5* and *Lgals3*. Using a zebrafish model of tailfin injury, we demonstrated that antisense morpholino-mediated knockdown of *blt2a* or chemical inhibition of BLT2 signaling impairs macrophage migration. We further replicated these findings in zebrafish models of islet injury and liver inflammation. Moreover, we established the applicability of our zebrafish findings to mammals by showing that macrophages of *Ltb4r2−/−* mice have defective migration during lipopolysaccharide stimulation *in vivo*. Collectively, our results demonstrate that BLT2 mediates macrophage migration during inflammation, which implicates it as a potential therapeutic target for inflammatory pathologies.

Inflammation is a conserved biological response that is stimulated by invading pathogens, injury, or systemic metabolic disease processes. Acutely, inflammation is terminated upon resolution of the underlying cause, but persisting inflammation can contribute to tissue pathologies that result in chronic disease processes, including type 1 and type 2 diabetes (T1D and T2D), non-alcoholic fatty liver disease (NAFLD), and cardiovascular diseases, among others (1). A major cell type contributing to tissue inflammation is macrophages, innate immune cells derived from the myeloid blood lineage (2). Within tissues, macrophages release inflammatory mediators (*e.g.* chemokines, cytokines, reactive oxygen species), clear dead/dying cells (efferocytosis), and take up and present antigens/neoantigens to the adaptive immune system (3). Acutely, some of these actions are adaptive, pro-resolving functions, but during chronic inflammation, these actions can lead to further tissue damage and maladaptive pathology. In this regard, depletion or functional inactivation of macrophages relieves the pathogenesis of various inflammatory diseases, including T1D, T2D, NAFLD, and rheumatoid arthritis (4, 5). However, because inactivation or depletion of macrophages would not be desirable owing to the essential roles they perform during innate immunity, identifying targets that mediate macrophage recruitment to the injured tissues during inflammation serves alternative therapeutic potential.

Lipoxygenases (LOX) are enzymes that catalyze the di-oxygenation of polyunsaturated fatty acids to generate signaling molecules. Specifically, 12-LOX catalyzes the conversion of arachidonic acid into the proinflammatory metabolite 12-hydroxyeicosatetraenoic acid (12-HETE) (6). In humans and mouse models, 12-LOX is expressed in several tissues, including pancreatic islets, hepatocytes, and macrophages, and its expression levels increase during tissue inflammation (7). Notably, 12-LOX activity in macrophages promotes their migration to injured tissues, which then initiates an increasingly pro-inflammatory environment (8). Depletion or inhibition of 12-LOX activity impairs the infiltration of tissues by macrophages and confers protection from the pathogenesis of T1D, T2D, NAFLD, and colitis (7).

Leukotriene B4 receptor 2 (also known as BLT2) is a G-protein-coupled receptor (GPCR) that has been described as a low-affinity receptor for 12-HETE as well as for several other pro-inflammatory metabolites, including the potent chemoattractant leukotriene B4 and eicosanoids 12(S)-HPETE and 15(S)-HETE (9). Despite these observations, the functions of BLT2 in inflammation and macrophage function during disease remain incompletely understood. In this study, we leveraged the genetic tractability of zebrafish and the mammalian platform of mice to investigate the role of BLT2 in macrophages in the setting of inflammation. Unbiased transcriptomics *in vitro* revealed a potential role for BLT2 in promoting macrophage migration, and tissue injury models *in vivo* confirmed the requirement for BLT2 in chemotactic function. Our data suggest that BLT2 is a potential contributor to inflammatory pathologies.

## Results

### BLT2 is expressed in metabolically active tissues and its loss does not impact glucose control

To investigate the roles of BLT2 in the setting of inflammation, we first analyzed its expression patterns in metabolic tissues and immune cells that might participate in inflammatory processes. We isolated tissues from C57BL/6 mice and quantified *Ltb4r2* mRNA (encoding BLT2) using quantitative reverse transcription-PCR (qRT-PCR). We detected *Ltb4r2* mRNA expression in the liver, spleen, adipose tissue, bone marrow-derived macrophages (BMDMs), and peritoneal macrophages (Fig. S1*A*). However, we detected little to no *Ltb4r2* expression in pancreatic islets, which are responsible for insulin secretion (Fig. S1*A*). Taken together with the role of BLT2 as a low-affinity receptor to a range of proinflammatory metabolites and chemoattractants, these findings suggest that BLT2 might play a role in macrophage function.

To investigate the role of BLT2 in macrophages, we studied *Ltb4r2−/−* mice that have been previously characterized and validated to globally lack BLT2 expression with no confounding reduction in the expression levels of the paralogue BLT1 (10). As *Ltb4r2* expression was detected in various metabolic tissues, we first queried if BLT2-deficiency would have adverse metabolic or developmental consequences. Body weight measurements, glucose and insulin tolerance tests demonstrated that both male and female *Ltb4r2−/−* mice exhibited overall normal growth and glucose tolerance that were indistinguishable from WT littermates (Fig. S1, *B*–*G*), indicating that *Ltb4r2−/−* mice are metabolically neutral without discernible metabolic defects.

### BLT2 governs macrophage migration

**Figure 1:**
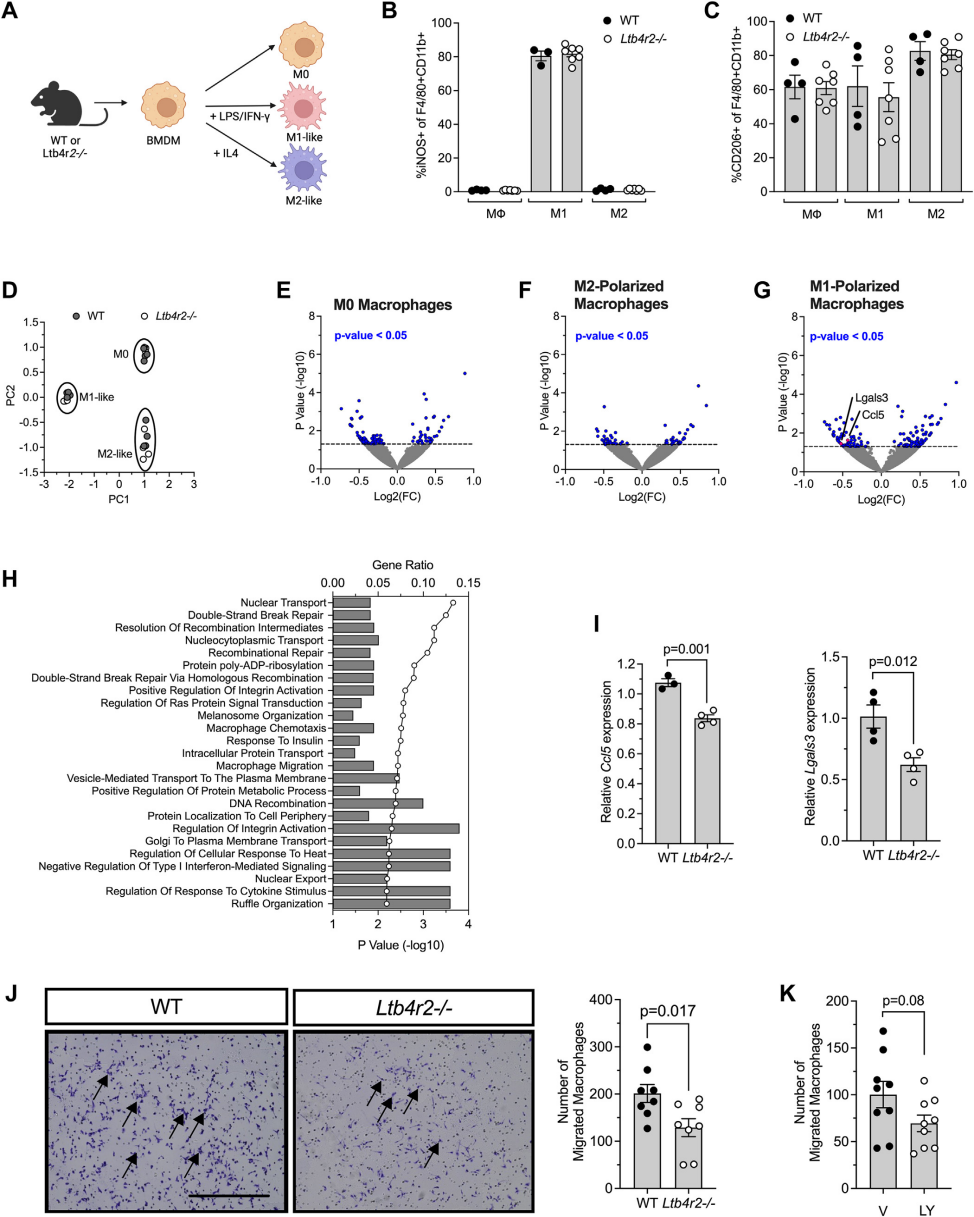
**Macrophages from *Ltb4r2−/−* mice exhibit reduced chemotaxis.** Bone-marrow-derived macrophages (BMDMs) were isolated from WT and *Ltb4r2−/−* mice and unpolarized (M0) or polarized to M2-like and M1-like states. *A*, schematic representation of experimental design; (*B*) iNOS+ cells as a percentage of F4/80+ CD11b+ cells as determined by flow cytometry; (*C*) CD206+ cells as a percentage of F4/80+ CD11b+ cells as determined by flow cytometry; (*D*) principal component analysis plot of RNA sequencing data; (*E*) Volcano plot showing differentially expressed genes by *p* value in M0 macrophages (each dot represents a distinct gene); (*F*) Volcano plot showing differentially expressed genes by *p* value in M2-polarized macrophages; (*G*) Volcano plot of differentially expressed genes by *p* value in M1-polarized macrophages; (*H*) Enrichr Gene Ontology pathway analysis of differentially expressed genes in M1-polarized macrophages, ordered by *p*-value; (*I*) relative expression of *Ccl5* and *Lgals3* in M1-like macrophages from WT and *Ltb4r2−/−* mice; (*J*) representative images of macrophage migration in transwell plates (*left panels*) and respective quantification (*right panel*). *Arrows* indicate macrophages (*blue*) in *left panel* images. Scale bar = 500 μm; (*K*) quantification of migrated macrophages after treatment with vehicle (V) or 6 μg LY255283 (LY). All data are presented as mean ± SEM and each data point represents an independent biologic replicate from different animals. For statistics, unpaired two-tailed t tests were used for comparison of two values, and 1-way ANOVA with Tukey post-test for more than two comparisons.

As BLT2 has been previously associated with proinflammatory signaling pathways (10), we first sought to determine if BLT2 governs the polarization of macrophages to a pro-inflammatory state. We isolated BMDMs from *Ltb4r2−/−* mice or their WT littermates and stimulated them with a combination of lipopolysaccharide (LPS) and interferon-γ (IFN-γ) to induce polarization into an “M1-like” proinflammatory state, or with interleukin-4 (IL-4) for polarization into an “M2-like” anti-inflammatory state (11) (Fig. 1*A*). We subsequently assessed polarization through flow cytometry using the pro-inflammatory M1 macrophage marker iNOS or the alternatively activated M2 macrophage marker CD206. We observed that WT and *Ltb4r2−/−* macrophages demonstrated indistinguishable propensity for both classical and alternative polarization (Fig. 1, *B* and *C*), indicating that BLT2 does not mediate cytokine-induced polarization of macrophages.

To query other potential roles of BLT2 in macrophage function in an unbiased manner, we performed bulk RNA-sequencing on BMDMs isolated from *Ltb4r2−/−* mice and their WT littermates that were unpolarized (M0) or polarized to M2-or M1-like states (using IL4 or lipopolysaccharide/IFN-γ, respectively). Principal component analysis of transcriptomes revealed that macrophages from the two genetic groups clustered by polarization, and replicates of each group clustered separately together (Fig. 1*D*). Pairwise comparison of gene expression levels between the WT and *Ltb4r2−/−* macrophages using a *p* < 0.05 cutoff revealed modest alterations of the transcriptome in each polarization group (M0, M2, and M1) (volcano plots in Fig. 1, *E*–*G*, and Tables S1–S3). While M0 and M2 states showed 100 and 68 differentially expressed genes, respectively, between the WT and *Ltb4r2−/−* groups (Fig. 1, *E* and *F* and Tables S1 and S2), we observed the largest differential gene expression in the pro-inflammatory M1 state with a total of 153 genes differentially expressed (Fig. 1*G* and Table S3), suggesting that BLT2 might play a role in mediating macrophage function during inflammation. Upon Gene Ontology pathway analysis using Enrichr (12), the M1-polarized *Ltb4r2−/−* macrophages showed alterations in pathways relating to macrophage chemotaxis and migration, among others (Fig. 1*H*). Importantly, genes that are mediators of macrophage migration under inflammatory conditions (*Ccl5, Lgals3*) (13, 14) were significantly downregulated in “M1-like” *Ltb4r2−/−* macrophages (Fig. 1*G*). We separately confirmed the reduction in the expression levels of these genes in *Ltb4r2−/−* BMDMs under M1 conditions by qRT-PCR (Fig. 1*I*). Taken together, the unbiased transcriptomic analyses suggested a potential role for BLT2 in mediating macrophage migration that might be relevant during tissue inflammation.

To determine the requirement for BLT2 in the context of macrophage migration, we next performed transwell migration assays *in vitro* using peritoneal macrophages isolated from *Ltb4r2−/−* mice and their WT littermates. We placed the isolated macrophages to the upper chamber of the chemotaxis system and loaded the bottom chamber with 10% FBS-RPMI media to stimulate their migration. After 4 h of incubation, we quantified the number of macrophages that have migrated to the lower chamber. We observed that the number of *Ltb4r2−/−* macrophages that migrated through the transwell membrane were significantly reduced compared to the number of WT macrophages (Fig. 1*J*), consistent with impaired migration in the absence of BLT2. To confirm that the observed phenotype results from the loss of BLT2 and not secondary effects that might have been introduced by the mutation at the *Ltb4r2* locus, we repeated the transwell migration experiments after treating the WT macrophages with the small molecule BLT2 antagonist LY255283 (15) or DMSO (as vehicle control). We observed a similar reduction in the number of macrophages that migrated through the transwell membrane upon treatment with LY255283 (Fig. 1*K*). These results, together with the transcriptomic analyses, suggest that BLT2 is important for mediating macrophage migration.

### Zebrafish ltb4r2a, but not ltb4r2b, mediates macrophage migration during tissue injury

**Figure 2:**
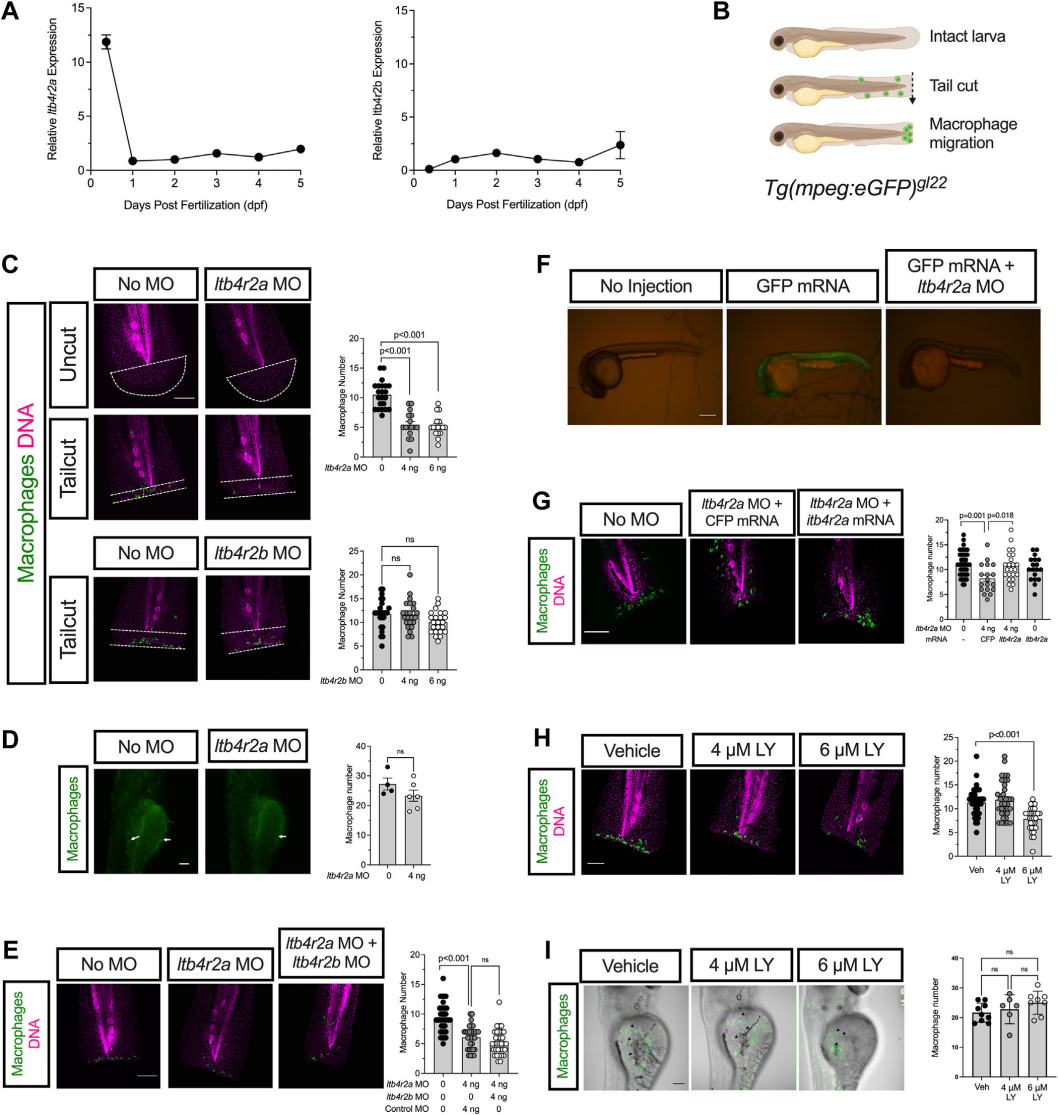
**Morpholino oligonucleotide against *ltb4r2a* in zebrafish impairs macrophage migration.***A*, qRT-PCR for *ltb4r2a* and *ltb4r2b* during different ages of zebrafish. N = 3 biological replicates per time point. *B*, schematic representation of tail cut injury in *Tg(mpeg:eGFP)gl22* zebrafish larvae in which macrophages are labeled *green*. *C*, representative images of macrophages (*green*) and DNA (*magenta*) (*left panels*) following injection of morpholino oligonucleotides (MOs) against *ltb4r2a* and *ltb4r2b,* and corresponding quantification of macrophage number at the site of injury (*right panels*). Scale bar = 100 μm; *D*, representative images of macrophages (*green*) in *Tg(mpeg:eGFP)gl22* zebrafish larvae yolk sac (*left panels*) and quantification of macrophage numbers (*right panel*). Scale bar 100 μm. *E*, representative images of macrophages (*green*) and DNA (*magenta*) in zebrafish larvae injected with indicated MOs (*left panels*) and quantification of macrophage number at the site of injury (*right panel*). Scale bar = 100 μm. *F*, representative images of 1 dpf zebrafish embryos following injection with GFP mRNA with or without the *ltb4r2a* MO. Scale bar = 500 μm. *G*, representative images of macrophages (*green*) and DNA (*magenta*) in zebrafish larvae injected with indicated MOs and mRNAs (*left panels*) and quantification of macrophage number at the site of injury (*right panel*). Scale bar = 100 μm. *H*, representative images of macrophages (*green*) and DNA (*magenta*) in zebrafish larvae treated with vehicle or indicated concentrations of LY255283 (*left panels*) and quantification of macrophage number at the site of injury (*right panel*). Scale bar = 100 μm. *I*, representative images of macrophages (*green,* indicated by *black arrowheads*) in *Tg(mpeg:eGFP)gl22* zebrafish larvae yolk sac following treatment with with vehicle or indicated concentrations of LY255283 (*left panels*) and quantification of macrophage numbers (*right panel*). Scale bar = 100 μm. Data are presented as mean ± SEM; Each data point represents an independent biological replicate from different animals. For statistics, unpaired two-tailed t-tests were used for comparison of two values, and 1-way ANOVA with Tukey post-test for more than two comparisons.

To determine the role of BLT2 in chemotaxis in settings of inflammation *in vivo*, we next utilized zebrafish models of tissue injury. Zebrafish have two genes encoding BLT2, *ltb4r2a,* and *ltb4r2b*, each with over 30% identity and 60% amino acid similarity to the human *LTB4R2* sequence (16). Moreover, developing zebrafish larvae (2–3 days post fertilization, dpf) have intact innate immune systems with *mpeg*-expressing macrophages (17), which makes zebrafish a tractable and applicable model system for the analysis of innate immune cells during inflammation (8, 18). To quantify the expression of zebrafish *Ltb4r2* orthologs during early developmental stages that are relevant to our study, we extracted total mRNA from whole zebrafish embryos at 9 h post fertilization (hpf) and 1 to 5 dpf timepoints and performed qRT-PCR. We observed that both *ltb4r2a* and *ltb4r2b* transcripts were expressed at all the investigated time points (Fig. 2*A*). Whereas the expression levels of *ltb4r2a* were the highest at 9 hpf and substantially decreased after 1 dpf, the expression levels of *ltb4r2b* were the lowest during the earliest time points and increased at 1 dpf (Fig. 2*A*), suggesting that the two orthologues might have different functions during development.

To query the roles of each *ltb4r2* gene in macrophage migration, we employed the well-characterized *Tg(mpeg:eGFP)*^*gl22*^ transgenic zebrafish line, wherein macrophages are labeled with the green fluorescent protein (GFP) (8, 17). We injected *Tg(mpeg:eGFP)*^*gl22*^ zygotes with translation-blocking morpholino oligonucleotides (MO) against each *ltb4r2* ortholog. At 3 dpf, we performed mechanical tailfin injury, which promotes rapid macrophage migration to the injury site as a part of the inflammatory response (19), and quantified the number of macrophages that have migrated to the site of injury at 6 h post injury (Fig. 2*B*). We observed no macrophages at the tailfins of control and MO-injected embryos in the absence of an injury (Fig. 2*C*), suggesting that the macrophage number observed at the tail cut sites reflects the extent of migration induced by the injury. Importantly, in the embryos injected with the *ltb4r2a* MO, we observed a significant reduction in the number of macrophages at the injury site compared to the control embryos (Fig. 2*C*). Macrophage numbers in tissues that were not injured were not affected by the *ltb4r2a* MO injection (Fig. 2*D*), suggesting that *ltb4r2a* MO does not affect macrophage production (only their migration to sites of injury). By contrast, we did not observe any difference in the macrophage numbers at the injury site upon *ltb4r2b* MO injection (Fig. 2*C*). To determine if there is a partial functional overlap between *ltb4r2a* and *ltb4r2b* that would cause the double knockdown phenotype to be more severe than either of the individual knockdowns, we next co-injected the *ltb4r2a* and *ltb4r2b* MOs and performed tailfin injury assays. The effect of the co-injection was not significantly different from the effect of *ltb4r2a* MO alone (Fig. 2*E*), further supporting that *ltb4r2b* does not play a role in mediating macrophage migration.

To ensure that the observed reduction in macrophage migration results from the specific knockdown of *ltb4r2a* levels rather than off-target effects of MO injection, we next confirmed the efficacy and specificity of the *ltb4r2a* MO. As there are no commercially available antibodies reactive against the zebrafish BLT2a protein, we confirmed the knockdown by an alternative approach. We generated a modified GFP mRNA by fusing the 25 nucleotide-long sequence targeted by the *ltb4r2a* MO to the 5′ end of the GFP coding sequence. Upon injection of the modified GFP mRNA into zygotes with no fluorescent transgenes, we observed ubiquitous green fluorescence throughout the embryos (Fig. 2*F*, *middle panel*). Co-injection of the *ltb4r2a* MO with the GFP mRNA eliminated any discernible GFP fluorescence (Fig. 2*F*, *right panel*), indicating that the *ltb4r2a* MO is effective in targeting mRNAs that contain its target sequence. Next, to address specificity of the *ltb4r2a* MO, we generated a synthetic *ltb4r2a* mRNA that does not contain the full MO target sequence. Whereas injection of the synthetic *ltb4r2a* mRNA alone did not have any effect on macrophage migration, co-injection of the mRNA with the *ltb4r2a* MO fully rescued the defect in migration (Fig. 2*G*). To verify the findings obtained with the *ltb4r2a* MO, we repeated the tailfin injury assays after treating the embryos with the BLT2 antagonist LY255283. Treatment with the inhibitor resulted in a significant reduction in macrophages at the sites of tailfin injury compared to vehicle-treated embryos (Fig. 2*H*), whereas it did not have any effect on macrophages in tissues that were not injured (Fig. 2*I*). These findings are consistent with and confirm the results obtained with the *ltb4r2a* MO. Taken together, our data indicate that zebrafish BLT2 encoded by *ltb4r2a* promotes macrophage migration during tissue injury, but that the enzyme isoform encoded by *ltb4r2b* may have a distinct, yet undefined, role.

### Ltb4r2a depletion impairs macrophage migration during islet and liver inflammation in zebrafish

**Figure 3:**
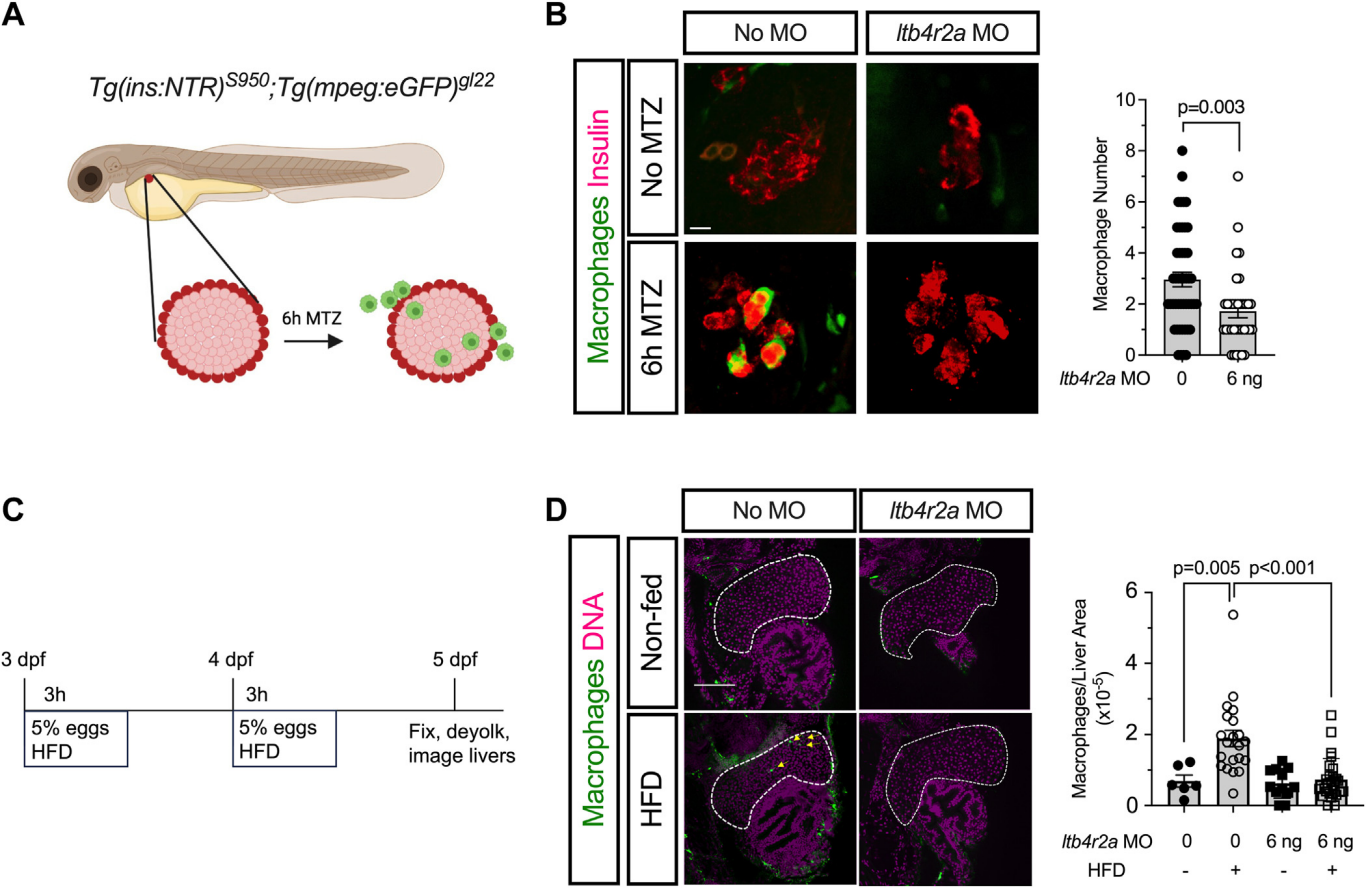
**Morpholino oligonucleotide against *ltb4r2a* in zebrafish impairs macrophage migration during β-cell and liver inflammation.***A*, schematic representation of the β-cell injury model using *Tg(ins:NTR)*^*S950*^*;Tg(mpeg:eGFP)*^*gl22*^ zebrafish expressing nitroreductase (NTR) in β cells and *green* fluorescent protein in macrophages. Double-transgenic larvae were treated with metronidazole (MTZ) for 6 h, followed by imaging of islets. *B*, representative images of macrophages (*green*) and insulin (*red*) following injection of morpholino oligonucleotide (MO) and treatment with MTZ, as indicated (*left panels*), and quantitation of macrophage number within islets (*right panel*). Scale bar = 10 μm. *C*, schematic representation of the liver injury model using *Tg(mpeg:eGFP)*^*gl22*^ zebrafish expressing green fluorescent protein in macrophages. Transgenic larvae were injected with or without MO at 0 to 1 h post fertilization, then incubated in 5% egg solution in egg water as indicated. *D*, representative images of macrophages (*green*) and DNA (*magenta*) and quantification of macrophage number in the liver. The *dotted line* indicates the outline of the liver parenchyma and *arrowheads* indicate macrophages (*green*) within the liver parenchyma. Scale bar = 100 μm. Data are presented as mean ± SEM; and each data point represents an independent biological replicate from different animals. For statistics, unpaired two-tailed t-tests were used for comparison of two values, and 1-way ANOVA with Tukey post-test for more than two comparisons.

We next sought to determine the relevance of BLT2-mediated macrophage migration in the setting of metabolic inflammation by utilizing zebrafish models of pancreatic islet and liver inflammation. We first utilized a well-characterized zebrafish islet β-cell injury model, in which transgenic zebrafish embryos that express the bacterial nitroreductase (NTR) enzyme under the β cell-specific insulin promoter *(Tg(ins:NTR)*^*s950*^*)* are treated with the prodrug metronidazole (MTZ) (20). The interaction between NTR and MTZ leads to the production of reactive oxygen species, which specifically causes β-cell stress, macrophage recruitment to the islets, and β-cell loss in a manner that reflects aspects of early T1D pathogenesis (Fig. 3*A*) (8, 21). Whereas we observed no macrophages within zebrafish islets in the absence of MTZ treatment, macrophages migrated into the islets with 6 h of MTZ exposure (Fig. 3*B*). Importantly, injection of the *ltb4r2a* MO led to a significant reduction in the number of macrophages within the islets upon MTZ treatment (Fig. 3*B*), consistent with the conclusion that *ltb4r2a* contributes to macrophage migration during islet inflammation.

In a second model of inflammation, we fed zebrafish embryos with a high-fat diet (HFD) consisting of a 5% homogenized egg solution at 3 and 4 dpf (Fig. 3*C*), which leads to lipid accumulation and a mild inflammatory state that promotes macrophage infiltration into the liver parenchyma (22). Consistent with prior studies, HFD feeding caused a significant increase in the number of macrophages observed within the liver boundaries (Fig. 3*D*) (22). Importantly, upon HFD-feeding, the number of macrophages per sampled liver area was significantly reduced in the embryos injected with the *ltb4r2a* MO compared to the non-injected controls (Fig. 3*D*), suggesting that *ltb4r2a* plays an essential role in mediating macrophage migration during liver inflammation. Together, these findings demonstrate that *ltb4r2a* contributes to macrophage migration in different settings of metabolic tissue inflammation.

### Ltb4r2 deletion suppresses macrophage infiltration during LPS-induced inflammation in mice

**Figure 4:**
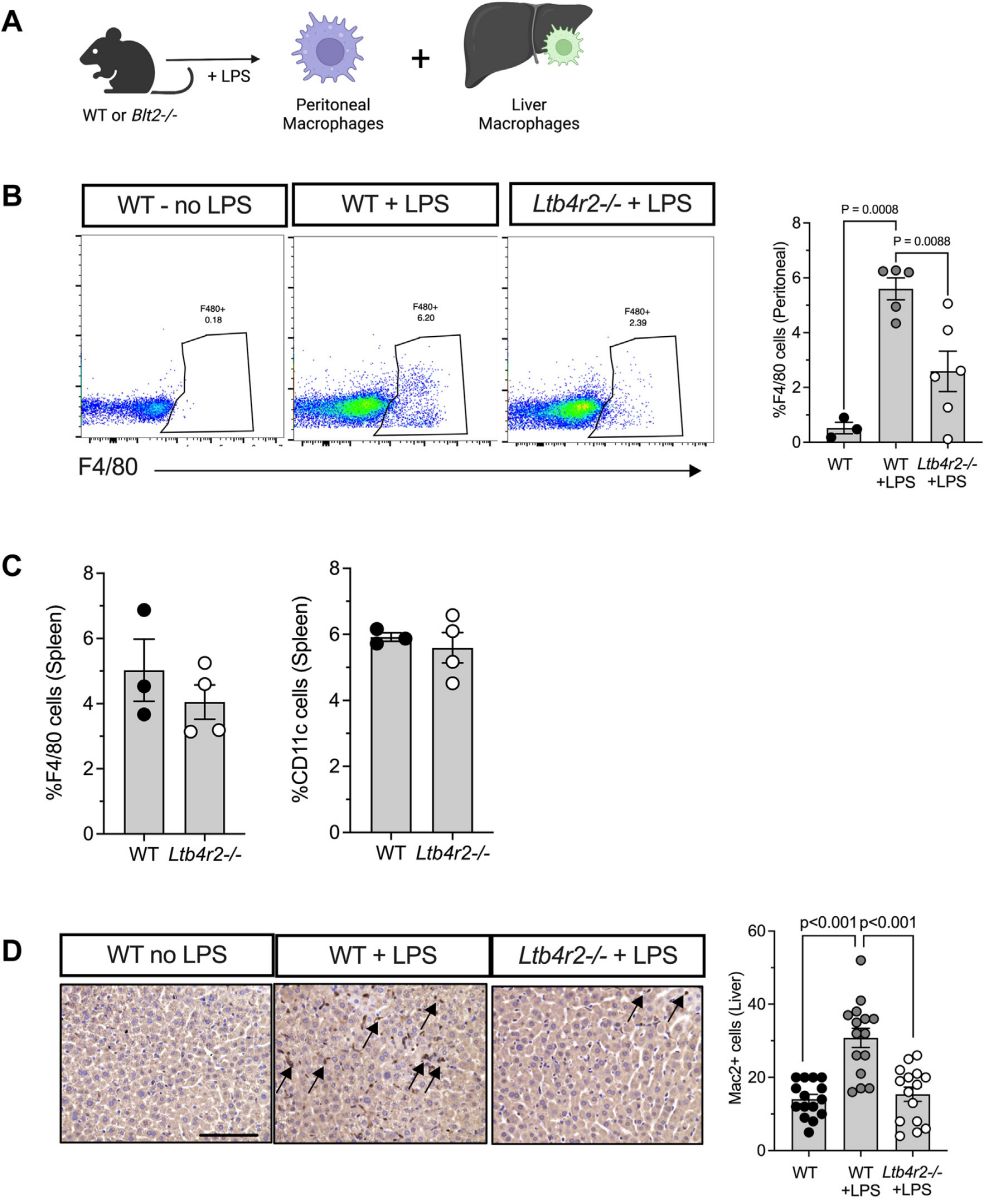
***Ltb4r2−/−* mice exhibit impaired macrophage migration following LPS-induced inflammation.***A*, schematic representation of LPS injury model in mice. WT and *Ltb4r2−/−* mice were injected with lipopolysaccharide (LPS), followed by quantitation of peritoneal macrophage number (by flow cytometry) and number of macrophages infiltrating the liver (by immunohistochemistry). *B*, representative flow cytometric dot plots of peritoneal F4/80+ cells (macrophages) (*left panels*) under the indicated conditions and corresponding quantitation (*right panel*). *C*, quantitation (from flow cytometry analysis) of F4/80+ and Cd11c+ cells from spleen in WT and *Ltb4r2−/−* mice following LPS injection. *D*, representative liver section images of Galectin 3+ macrophages (*blue*, indicated by *arrows*) under the indicated conditions (*left panels*) and quantification of macrophage number in liver tissue (*right panel*). Scale bar = 100 μm. Data are presented as mean ± SEM; ∗*p* < 0.05, and each data point represents an independent biological replicate from different animals. For statistics, 1-way ANOVA with Tukey post-test was used for comparisons.

Next, we assessed the role of BLT2-mediated macrophage migration in a mammalian inflammation model *in vivo*. To mimic a general state of inflammation (sepsis), we injected *Ltb4r2−/−* mice or their WT littermates with bacterial LPS intraperitoneally (23), then monitored the accumulation of peritoneal and liver macrophages 6 h later (Fig. 4*A*). We observed that treatment of the WT mice with LPS induced a marked increase in the number of F4/80+ peritoneal macrophages as assessed by flow cytometry (Fig. 4*B*). In *Ltb4r2−/−* mice compared to WT littermates, the number of peritoneal macrophages induced upon LPS-treatment was significantly lower (Fig. 4*B*), indicating that loss of BLT2 suppresses peritoneal macrophage accumulation during inflammation. Notably, the number of circulating white blood cells and the percentages of circulating monocytes (the precursors to tissue macrophages) were unaltered in *Ltb4r2−/−* compared to WT littermates before and after LPS injection (Table 1). Additionally, the numbers of F4/80+ and CD11c+ macrophages in the spleen following LPS injection were also unaltered in *Ltb4r2−/−* mice (Fig. 4*C*). These latter findings confirm that the reduction in peritoneal macrophages following LPS injection in *Ltb4r2−/−* was not a result of global declines in macrophage numbers.

**Table 1:** Complete blood count parameters in WT and Ltb4r2−/− mice

Cell type	Before LPS treatment			After LPS treatment		
	WT (n = 3)	Ltb4r2−/− (n = 4)	*p*-value	WT (n = 3)	Ltb4r2−/− (n = 4)	*p*-value
	Mean ± SD	Mean ± SD		Mean ± SD	Mean ± SD	
White Blood Cell number (x10^3^/μl)	7.78 ± 1.55	8.55 ± 1.74	0.5545	3.37 ± 0.61	3.91 ± 1.58	0.6055
Neutrophil (%)	16.86 ± 2.65	15.78 ± 4.06	0.6978	40.06 ± 3.55	46.45 ± 16.47	0.5474
Lymphocyte (%)	70.2 ± 6.85	72.86 ± 8.96	0.677	42.16 ± 3.97	37.8 ± 11.86	0.5747
Monocyte (%)	9.26 ± 5.31	7.38 ± 4.12	0.5914	14.33 ± 2.19	11.07 ± 4.47	0.3048
Eosinophil (%)	3.66 ± 1.92	3.98 ± 1.09	0.7734	3.43 ± 0.65	4.67 ± 1.49	0.2427

Because LPS stimulation is implicated in promoting inflammatory cell infiltration in the liver and has been used to model acute and chronic liver inflammation in mouse models (24, 25), we next quantified the Mac2 (encoded by *Lgals3*)-positive macrophages in liver sections by immunohistochemistry. We observed that loss of BLT2 resulted in reduced accumulation of macrophages (Fig. 4*D*), confirming the role of BLT2 in mediating macrophage migration during liver inflammation in mammalian models. Collectively, our findings suggest that BLT2 is required for mediating macrophage recruitment and infiltration in mammalian settings of inflammation.

## Discussion

GPCRs are transmembrane proteins that mediate cellular response to extracellular stimuli. Among these GPCRs, BLT2 appears to function as a low-affinity receptor to a variety of lipid signaling molecules, including leukotriene B4, 12(S)-HETE (9), and 12-HHT (26) that are associated with predominantly proinflammatory functions and have been implicated as therapeutic targets for various inflammatory conditions (7, 26, 27). Although BLT2 as a common downstream receptor for these ligands makes it a promising target for inflammatory pathologies, its precise role(s) during inflammation remains incompletely understood. Two prior studies that addressed this issue implicated BLT2 as a contributor to auto-antibody-driven inflammatory arthritis (10) and airway inflammation (28). However, to date, the general roles BLT2 might play during inflammation and the mechanistic basis and specific cellular origins for these roles remain uncharacterized. Using a combination of expression and transcriptional analysis alongside injury models *in vivo* involving genetic knockout/silencing and small-molecule-based inhibition approaches, we show that BLT2 promotes macrophage migration to injured tissues during inflammation. Moreover, the viability and health of *Ltb4r2−/−* mice further suggest that systemic inhibition of BLT2 might prove a viable strategy for the treatment of disorders associated with inflammation.

Previous studies have reported *Ltb4r2* mRNA expression in various tissues of mice, including mast cells (29), small intestine, skin, colon, and spleen (30). In one study (30), *Ltb4r2* expression was not detected in macrophages by Northern blot. Our mRNA expression analysis in mice demonstrates the presence of *Ltb4r2* mRNA in metabolic tissues such as the liver, adipose, and macrophages. Given the relatively low abundance of GPCRs, we attribute the difference in our results to the much greater sensitivity of the method we employed for mRNA detection (qRT-PCR) compared to the Northern blot. Although the expression levels of *Ltb4r2* in macrophages were lower relative to the other tissues we investigated, our study nevertheless revealed that BLT2 is an essential contributor to macrophage function. Notably, our transcriptomic analysis revealed that *Ltb4r2−/−* macrophages had altered gene expression signatures with a downregulation in key transcripts (*Ccl5, Lgals3*), essential mediators of macrophage migration. Moreover, the most prominent alterations were observed in M1 macrophages, suggesting that BLT2 might be crucial in shaping macrophage function during inflammation.

Our zebrafish studies that utilized different models of tissue inflammation further support the relevance of BLT2 in inflammatory conditions. Zebrafish larvae offer the advantage of an intact innate immune system. The genetic and physiological similarities between the hepato-pancreatic systems of mammals and zebrafish make them suitable model organisms for studying metabolic conditions. Importantly, zebrafish models have been extensively utilized to study the dynamics of macrophage migration (17, 31) and model islet and liver inflammation (8, 22, 32). Leveraging the well-characterized tailfin injury model and two distinct models of metabolic inflammation, our study demonstrated that zebrafish *Ltb4r2a* (but not *Ltb4r2b*) promotes macrophage migration during different inflammatory settings, indicating its role as a contributor to inflammatory responses. Our ability to replicate these findings in mouse models *in vivo* further highlights the applicability of this proinflammatory chemotactic role of BLT2 in mammals. Moreover, our observation that depleting BLT2 or impairing its activity (*via* a small molecule inhibitor) in distinct settings of tissue inflammation impairs macrophage infiltration further supports the notion that BLT2 within macrophages mediates these chemotactic effects.

Our findings do not exclude the possibility that BLT2 in other tissues might play independent or related roles during inflammation. GPCRs are a diverse group of membrane proteins that associate with heterotrimeric G-proteins, consisting of G_α_, G_β_, and G_γ_ subunits. Interaction between the GPCR and its ligand induces a conformational change that activates the subunits and causes their dissociation from the GPCR to lead to a cascade of signaling events in the cell (33). As these signaling events can vary based on the activating ligand and the cellular context (34, 35), it is possible that the tissue-specificity of BLT2 expression or ligand production may dictate BLT2 function. Consistent with this idea, prior studies have reported that BLT2 expression in the cryptic colon cells might be protective against inflammatory colitis (36) and that BLT2 expression in the skin keratinocytes might be necessary for epidermal wound healing (37). We believe that these findings that implicate BLT2 as an anti-inflammatory mediator with pro-resolving functions result from the difference in the cellular source of BLT2. Although further studies will be needed to resolve these differences, our results reveal the importance of considering the disease setting and cellular source of BLT2 into account when contextualizing its functional role. Together, our findings provide evidence that the BLT2 receptor is an important contributor to macrophage migration during tissue inflammation. While this role, conserved between zebrafish and mammals, implicates BLT2 as a contributor to inflammatory pathologies, further studies will be needed to determine its downstream mediators.

## Experimental procedures

### Animals

All mice and zebrafish used in this study were housed under specific pathogen-free conditions with a standard 12h light: dark cycle for mice and 14:10 light: dark cycle for zebrafish. All mouse and zebrafish experiments were approved by the University of Chicago Institutional Animal Care and Use Committee. *Ltb4r2−/−* mice on *C57BL/6J* background were purchased from Jackson Laboratories and subsequentially bred in-house.

Zebrafish were maintained in 2 L tanks at 28.5 °C in a recirculating aquaculture system. The transgenic lines used for experiments include *Tg(mpeg:eGFP)*^*gl22*^ (17) and *Tg(ins:NTR)*^*S950*^ (20). Embryos were collected at spawning and placed into egg water (0.1% Instant Ocean salts, 0.0075% calcium sulfate, and 0.1% methylene blue). At 24 hpf, the media was replaced by egg water supplemented with 0.003% 1-Phenyl-2-thiouria (PTU) to prevent pigmentation.

### Procedures involving mice

For GTTs, mice were fasted for 16 to 18 h animals were intraperitoneally injected with glucose (1 g/kg) and blood glucose was measured at 0, 10, 20, 30, 60, 90, and 120 min using an AlphaTrak glucometer. For ITTs, 0.75 U/kg of insulin was injected IP after 2 h fasting and blood glucose was measured at 0, 15, 30, 45, and 60 min. For studies involving LPS injections, Male and female 11-week-old WT and *Ltb4r2−/−* mice received intraperitoneal injection of *E. coli* (O111:B4) LPS at a concentration of 5 mg/kg or PBS. Adipose tissue, spleen, and peritoneal macrophages were harvested after 6 h. For measurement of complete blood count, blood samples were collected from the tail vain of 8 to 10 week-old WT and *Ltb4r2−/−* mice in EDTA capillary vials (Kent Scientific Corporation) and analyzed by an automated hematology analyzer (Heska Element HT5).

### Procedures involving zebrafish

For experiments involving morpholino oligonucleotides (MOs), MOs were purchased from GeneTools LLC with the following sequences: *Ltb4r2a*: 5′-ACAGAAGGTTCAACGCCATCTTGGC-3′; *Ltb4r2b*: 5′-AGCTGCCATTTTCCAATGCCATTTG-3′. For microinjections, morpholinos or mRNAs were diluted with nuclease free water and mixed with 0.1% of phenol red marker. The injection volume was calibrated by determining the diameter of the injection droplet on mineral oil to inject 4, 6, or 8 ng of the MOs or 50 to 200 pg of the mRNAs. Zygotes were collected at spawning and injected at the 1-cell stage. The embryos that are marked with phenol red were sorted for use in later experiments.

For experiments involving mRNA injections, capped and poly-A-tailed mRNAs were obtained from the purified PCR products through *in vitro* transcription using the mMessage mMachine T7 Ultra kit (Thermo Fisher). The mRNAs were then purified through Lithium Chloride precipitation, and concentrations were determined using NanoDrop. The mRNAs were visualized on 1% agarose gel treated with 1.5% bleach to ensure quality before injections (38).

For tailfin injuries, procedures followed those previously described (19). Briefly, 3 dpf larvae were paralyzed with 0.01% tricaine to prevent movement. The distal tips of the tailfins were cut with a sharp scalpel. Following injury, embryos were placed in a 28.5 °C incubator for 6 h. For islet injury assays, 3 dpf embryos were treated with 7.5 mM of MTZ in egg water or egg water alone, for 6 h. Liver inflammation studies followed previously detailed methods (22). 3 dpf larvae were incubated in 4 ml of 5% chicken egg solution for 3 h in dark at 28.5 °C and washed following feeding. Feeding was repeated at 4 dpf, and embryos were left to rest overnight following feeding. At the end of the specified incubation periods, the embryos were fixed with 3% formaldehyde in PEM buffer (100 mM PIPES, 1 mM MgSO_4_, 2 mM EGTA) at 4 °C for 18 h, washed, deyolked, and immunostained as previously described (39). The following antibodies were used for staining: guinea pig anti-insulin (Invitrogen, 1:100), chicken anti-GFP (Aves labs; 1:1000), TO PRO3 (Thermo Fisher; 1:1000), DAPI (Thermo Fisher; 1:2000). Secondary antibodies were used at 1:1000 dilution. The specificities of the antibodies were confirmed in tissue sections in which the primary or secondary antibody were omitted, and no relevant signal was observed. The stained embryos were mounted on glass slides with VECTASHIELD mounting medium (Vector Labs), and confocal imaging was performed using a Nikon A1 microscope, 20× objective. All image analyses and area measurements were done with the ImageJ software. For analysis of macrophage migration in tailfin injury assays, macrophage number between the cut site and the distal tip of the notochord rod was determined. In islet injury assays, the entire islet was imaged as a stack and the total macrophage number within the islet was quantified. In the liver inflammation studies, 18 liver slices were imaged; macrophage counts within the liver parenchyma were determined and divided by the total imaged liver area. For quantifying macrophages within the yolksacs following MO injection or treatment with the inhibitor, the embryos were mounted sideways in slides, and the GFP+ macrophages present on the visible side of the yolk were quantified.

For inhibitor studies, LY255283 was purchased from Tocris and dissolved in DMSO. 4 to 6 μM of LY255283 or an equal volume of DMSO were directly added into egg water supplemented with 0.003% PTU. Embryos were pre-treated with LY255283 or DMSO for 30 min before tailfin injury and the treatment was continued after the injuries for 6 h.

### Macrophage isolation and experiments

Peritoneal macrophages were isolated from 11-week-old mice by injecting 10 ml ice-cold PBS containing 3% FBS into the peritoneal cavity and removed using a 20-gauge needle as described previously (40). Cells were lysed with RBC lysis buffer to remove red blood cells and washed with PBS. Isolated peritoneal macrophages were used for flow cytometry analysis. For polarization studies *in vitro*, bone marrow-derived macrophages (BMDMs) were isolated as described previously (8) and cultured for 7 days in a complete medium (RPMI containing 10% FBS, 10 mM HEPES, and 100 U/ml penicillin/streptomycin) supplemented with 10 ng/ml M-CSF. On day 7, BMDM were stimulated with 10 ng/ml LPS and 25 ng/ml IFN-γ for M1-like polarization and 10 ng/ml IL-4 for M2-like macrophage polarization for 16 h. To stain for surface antigens, cells were incubated with F4/80 (Biolegend) and CD11b (Biolegend) antibodies for 20 min on ice. For intracellular staining, cells were permeabilized with Cytofix/Cytoperm (BD Pharmigen) and incubated with CD206 (Biolegend) and iNOS (Invitrogen) antibodies for 20 min on ice. All antibodies were used as 1:100 dilution. For immune profiling studies of macrophages from spleen, spleens were harvested as a single cell suspension in buffer containing 10% FBS in PBS from LPS-treated WT and *Ltb4r2−/−* mice. Cells were filtered through 70 μm filter, and red blood cells were removed using RBC lysis buffer (Biolegend). Cells were analyzed on the Attune N x T Flow Cytometer (Thermo Fisher). Data were analyzed by FlowJo software (BD Biosciences). The specificities of the antibodies were confirmed in flow cytometry experiments in which the primary or secondary antibodies were omitted, and gating was performed to ensure no background signal.

For the migration assays, BMDMs (2 × 10^5^) were placed in the upper chamber of 8 μm transparent PET membrane (Corning), and RPMI containing 10% FBS was loaded in the bottom chamber. Cells were incubated for 4 h at 37 °C, fixed in formalin and stained with crystal violet. Migrated cells were mounted on a slide and imaged using a BZ-X810 fluorescence microscope (Keyence). The number of macrophages was quantified by manual counting by an observer blinded to sample identity.

### Immunohistochemistry

Adipose and liver tissues were fixed in 4% paraformaldehyde, paraffin-embedded, and sectioned. Tissues were immunostained with anti-F4/80 (Sigma: D2S9R; 1:150 dilution) or anti-Galectin 3 (Thermo Fisher: 14-5301-85 1:150 dilution) primary antibody followed by conjugated anti-rabbit Ig (Vector Laboratories) secondary antibody, DAB peroxidase substrate kit (Vector Laboratories), and counterstained with hematoxylin (Sigma). Images were acquired using a BZ-X810 fluorescence microscope (Keyence). The specificities of the antibodies were confirmed in tissue sections in which the primary or secondary antibody were omitted, and no relevant signal was observed.

### RNA isolation, quantitative reverse-transcription PCR, and RNA sequencing

Mouse: RNA was isolated from mouse tissues and macrophages using an RNAeasy Mini kit (Qiagen) and cDNA synthesis was performed using High-Capacity cDNA Reverse Transcription kit (Applied Biosystems) according to manufacturer’s instructions. Quantitative PCR was performed using Bio-Rad CFX Opus with a predesigned Taqman assay probe for mouse genes: *Ltb4r2*: Mm01321172_s1; *Ccl5*: Mm01302427_m1; *Lgals3*: Mm00802901_m1; and *Actb*: Mm01205647_m1 (Invitrogen). Relative gene expression was calculated using the comparative threshold cycle value (Ct) and normalized to *Actb*.

Zebrafish: Total mRNA was extracted from 9 hpf and 1 to 5 dpf zebrafish embryos using Trizol (Thermo Fisher) and subsequent phenol chloroform extraction as described (41). The isolated mRNA was used to prepare cDNA libraries using High-Capacity cDNA Reverse Transcription kit (Applied Biosciences), and quantitative PCR was performed using SensiFast Real-Time PCR kit (Bioline). The average Ct values were determined, normalized to the Ct values of *elfa*, and analyzed across time points by using the ΔΔCt method as previously described (42). The following primers were used for quantitative PCR analysis: *ltb4r2a* forward: 5′-GAGCGGGTTCAGAAAACACTG-3′; *ltb4r2a* reverse: 5′-TTCAACGCCATCTTGGCAAC-3′; *ltb4r2b* forward: 5′-CGCCTCAGACAGACCAAGTT-3′; *ltb4r2b* reverse: 5′-AGTAGACGTAACAGCACGGC-3′; *elfa* forward: 5′-CTTCTCAGGCTGACTGTGC-3′; *elfa* reverse: 5′-CCGCTAGCATTACCCTCC-3′.

For RNA sequencing experiments, macrophages were polarized as described above and RNA was isolated using RNAeasy Mini kit (Qiagen). Raw sequencing data was analyzed by Rosalind (https://rosalind.bio/), with a HyperScale architecture developed by Rosalind. Reads were trimmed using cutadapt (43). Quality scores were assessed using FastQC2. Reads were aligned to the *Mus musculus* genome build mm10 using STAR3. Individual sample reads were quantified using HTseq4 and normalized *via* Relative Log Expression (RLE) using DESeq2 R library5. DEseq2 was used to calculate fold changes and *p*-values and perform optional covariate correction. Enrichment was calculated relative to a set of background genes relevant for the experiment. To identify altered pathways, genes that are significantly altered between M1 WT and *Ltb4r2−/−* macrophages were loaded to Enrichr, and the resulting pathways were ordered by *p* value.

### Statistical analysis

Data analyses were performed using the GraphPad Prism 10 software package. Significant differences between the mean values were determined using a Student’s *t* test, where 2 means were compared, and 1-way ANOVA followed by post hoc Tukey’s test when more than 2 means were compared. The differences were considered statistically significant at *p* < 0.05.

## Data availability

Some of the data generated and analyzed in the current study (images) are not publicly available but are available from the corresponding author upon reasonable request. Original data included in this manuscript and statistical analyses are available at Mendeley Data (https://doi.org/10.17632/vpynbgwkh3.1). RNA sequencing datasets have been deposited to the NIH Gene Expression Omnibus (GEO) with the accession number GSE 242723.

## Supporting information

This article contains supporting information.

## Conflict of interest

The authors declare that they have no conflicts of interest with the contents of this article.
