# Supplementary material for: Leukotriene B4 receptor 2 governs macrophage migration during tissue inflammation
Source: J Biol Chem. 2023 Dec 12;300(1):105561. doi: 10.1016/j.jbc.2023.105561 (PMC10790086; doi:10.1016/j.jbc.2023.105561)
Supplement: Supporting Figure S1 [file mmc4.docx]

**
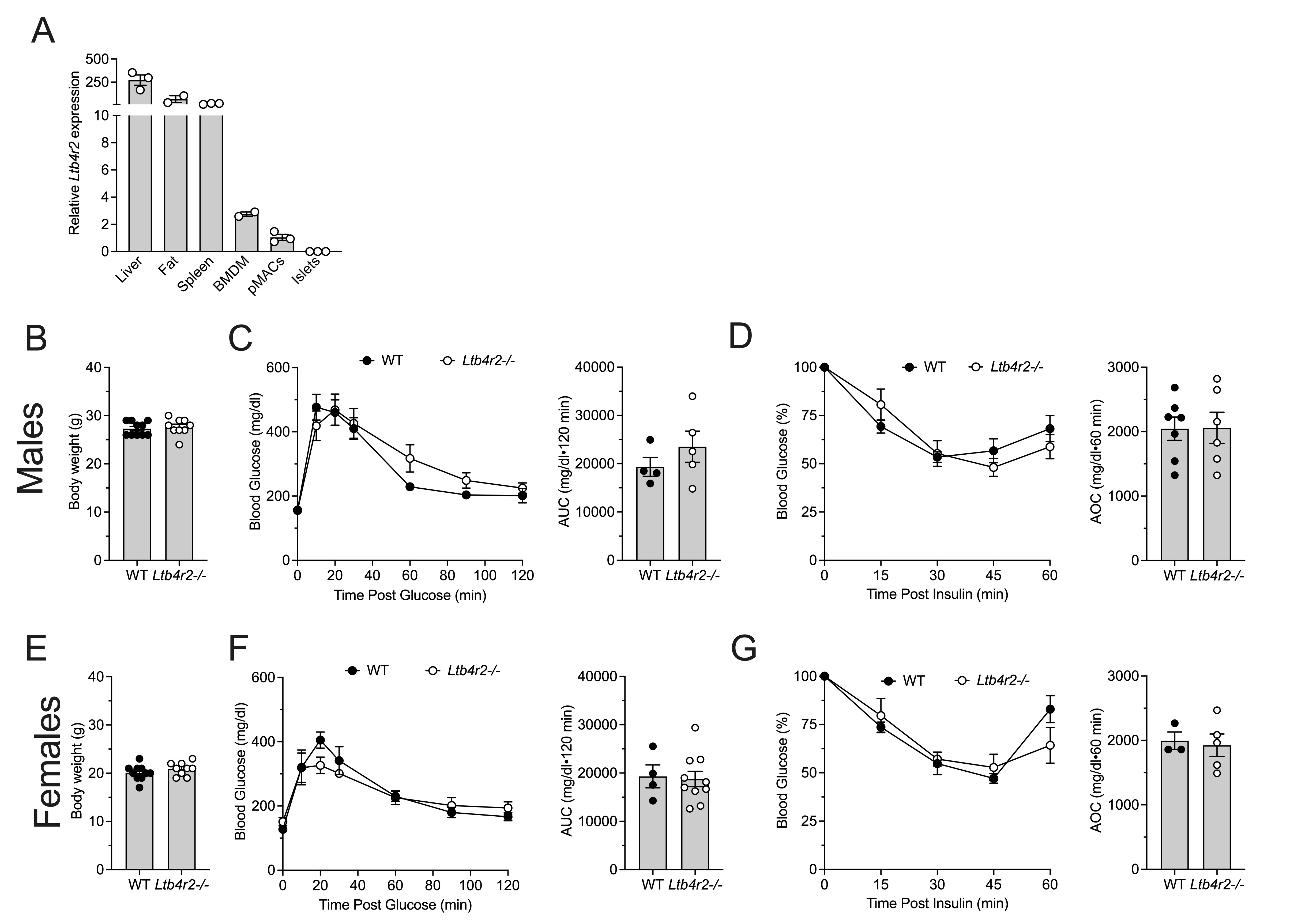
**

**Supplemental Figure S1: *Ltb4r2-/-* mice have normal glucose and insulin tolerance.** (***A***) Relative *Ltb4r2* gene expression in different B57BL/6J mouse tissues. (***B***) Body weights of 12-week-old male mice. (***C***) Glucose tolerance test (*left panel*) and corresponding area under the curve analysis (*right panel*) of 12-week-old male mice. (***D***) Insulin tolerance test (*left panel*) and corresponding area over the curve analysis (*right panel*) of 12- week-old male mice. (***E***) Body weights of 12-week-old female mice. (***F***) Glucose tolerance tests (*left panel*) and corresponding area under the curve analysis (*right panel*) of 12-week-old female mice. (***G***) Insulin tolerance test (*left panel*) and corresponding area over the curve analysis (*left panel*) of 12- week-old female mice. Data are presented as mean ±SEM; *P< 0.05, and each data point represents an independent biologic replicate from 4-10 different animals.
